# Nicotinamide Mononucleotide Attenuates Renal Interstitial Fibrosis After AKI by Suppressing Tubular DNA Damage and Senescence

**DOI:** 10.3389/fphys.2021.649547

**Published:** 2021-03-23

**Authors:** Yan Jia, Xin Kang, Lishan Tan, Yifei Ren, Lei Qu, Jiawei Tang, Gang Liu, Suxia Wang, Zuying Xiong, Li Yang

**Affiliations:** ^1^Department of Nephrology, Peking University Shenzhen Hospital, Shenzhen Peking University-The Hong Kong University of Science and Technology Medical Center, Shenzhen, China; ^2^Key Laboratory of Renal Disease, Renal Division, Department of Medicine, Peking University First Hospital, Peking University Institute of Nephrology, Ministry of Health of China, Beijing, China; ^3^Laboratory of Electron Microscopy, Pathological Center, Peking University First Hospital, Beijing, China

**Keywords:** AKI, NMN, DNA damage, senescence, renal fibrosis

## Abstract

Acute kidney injury (AKI) is a worldwide health problem currently lacking therapeutics that directly promote renal repair or prevent the occurrence of chronic fibrosis. DNA damage is a feature of many forms of kidney injury, and targeting DNA damage and repair might be effective strategies for kidney protection in AKI. Boosting nicotinamide adenine dinucleotide (NAD^+^) levels is thought to have beneficial effects on DNA damage repair and fibrosis in other organs. However, no kidney-related studies of such effects have been performed to date. Here, we have shown that NMN (an NAD^+^ precursor) administration could significantly reduce tubular cell DNA damage and subsequent cellular senescence induced by hydrogen peroxide and hypoxia in human proximal tubular cells (HK-2 cells). The DNA damage inhibition, antiaging and anti-inflammatory effects of NMN were further confirmed in a unilateral ischemia-reperfusion injury (uIRI) mouse model. Most importantly, the antifibrosis activity of NMN was also shown in ischemic AKI mouse models, regardless of whether NMN was administered in advance or during the recovery phase. Collectively, these results suggest that NMN could significantly inhibit tubular cell DNA damage, senescence and inflammation. NMN administration might be an effective strategy for preventing or treating kidney fibrosis after AKI.

## Introduction

Acute kidney injury (AKI) is a worldwide health problem characterized by sudden impairment of kidney function as a result of a toxic or ischemic insult. It is very common in the clinic, affecting 25–45% of high-risk hospitalized patients, such as surgery, trauma and intensive care unit patients (Rewa and Bagshaw, 2014). The mortality rate of severe AKI patients reaches 50%, while approximately 20–50% of surviving patients develop chronic kidney disease (CKD), and approximately 3–15% progress to end stage renal disease (ESRD) (Ferenbach and Bonventre, 2015; Varrier et al., 2015; Yang et al., 2015). However, no approved therapeutics have been directly indicated to promote renal repair or to prevent the occurrence of chronic fibrosis yet, and studying the molecular mechanism of AKI progression to CKD and finding targets of intervention are urgently needed.

Renal proximal tubular epithelial cells (PTECs), the most prominent cell type in the renal cortical tubulointerstitium, are particularly sensitive to injury (Bonventre and Yang, 2011). While the pathogenesis of AKI is multifactorial, recent studies have shown that DNA damage in PTECs plays an important role in the progression of AKI to CKD (Yang et al., 2010; Ferenbach and Bonventre, 2015; Canaud et al., 2019; Kishi et al., 2019). Maintaining and guaranteeing the DNA integrity of renal tubular epithelial cells may protect their structure and function after AKI. Nicotinamide adenine dinucleotide (NAD^+^) is a cellular metabolite in all living cells that is critical for fundamental biological processes, namely, DNA repair and energy metabolism. Since renal tubular cells are highly metabolically active, they are very sensitive to NAD^+^ depletion and impairment of ATP production. Replenishment of NAD^+^ levels via administration of its precursors, such as nicotinamide riboside (NR), nicotinamide mononucleotide (NMN), and nicotinamide (NAM), has been demonstrated to display beneficial effects against fibrosis and age-related diseases (Mills et al., 2016; Pham et al., 2019; Zheng et al., 2019). Multiple lines of evidence suggest that NMN might have important roles in protecting against DNA damage and ameliorating the long-term profibrotic response following AKI. However, to our knowledge, no studies have yet demonstrated such effects.

In our study, we first demonstrated that hydrogen peroxide and hypoxia resulted in DNA damage and subsequent G2/M arrest and senescence in HK-2 cells. NMN could decrease these injury phenotypes. Furthermore, we confirmed the DNA damage inhibition and antiaging effects of NMN administration in ischemic AKI mouse models. The antifibrosis ability of NMN was also proven in ischemic AKI mouse models, regardless of whether it was administered in advance or during the recovery phase. These findings have high translational potential as a pharmacologic strategy for improving fibrosis after AKI.

## Materials and Methods

### Cell Culture and Treatment

Human kidney-2 (HK-2) cells was cultured in Dulbecco’s modified Eagle’s medium (Gibco) with 10% fetal bovine serum (Gibco) at 37°C in a humidified 5% CO_2_ atmosphere. To induce injury *in vitro*, HK-2 cells were seeded in 6-well plates at 1 × 10^6^ cells/well and then were stimulated with 1 mM hydrogen peroxide (H_2_O_2_, Beijing Chemical Works, A1029005) (Lee et al., 2006; Small et al., 2014) and 1% O_2_ (Zhu et al., 2019) to generate a hypoxic environment using Whitley H35 hypoxystation (Don Whitely Scientific). Nicotinamide mononucleotide (β-NMN; Sigma-Aldrich; N3501) was dissolved in PBS and preserved at −20°C until use.

### Animal Models

Male C57BL/6 mice were purchased from SPF (Beijing) Biotechnology Co., Ltd. They were maintained on a 12:12 h light-dark cycle in a temperature-controlled room and were allowed free access to standard rodent chow and water. All animal studies were approved by the institutional Animal Care and Use Committee of Peking University First Hospital.

Warm ischemia was modeled by generating a unilateral ischemia-reperfusion injury (uIRI) in 8- to 10-week-old C57BL/6 mice. Briefly, mice were kept on a homeothermic unit and subjected to flank incisions. The left renal pedicle was exposed and clamped for 30 min. After removal of the clamp, the color of the kidneys turned from dark purple to pink. To examine the effect of NMN (β-NMN, Sigma-Aldrich, N3501) administration at the acute phase of uIRI, NMN (500 mg/kg body wt) (Guan et al., 2017; Li et al., 2017) or an equivalent amount of PBS was administered 20 min before the procedure by intraperitoneal injection and on days 1, 2, and 3 after surgery. Mice were euthanized at two time points: 4 h after the last injection (day 3) and day 21 after surgery (day 21), and then kidneys were collected from both sides. To examine the effect of NMN administration at the recovery phase of uIRI, NMN (500 mg/kg body wt) or an equivalent amount of PBS was administered intraperitoneally on days 3 and 14, and then mice were euthanized on day 21 after surgery, and kidneys were collected from both sides.

### NAD^+^ Measurement

NAD^+^ levels of HK-2 cells and kidney tissues was measured with an NAD/NADH Quantification Kit (Beyotime, S0175) according to manufacture’s instructions.

### Flow Cytometry

For cell cycle analysis, HK-2 cells were trypsinized and then washed with PBS for two times. After fixed in 1 mL of ice-cold 75% ethanol at 4°C overnight, HK-2 cells were incubated with 500 μL of PI/RNase staining buffer (BD PharMingen, BD 550825) for 15 min at room temperature. Cell cycle analysis was performed by flow cytometry using a BD FACSCalibur and analyzed with ModFit LT software. For analysis of DNA damage, HK-2 cells were incubated with a γH2A.X (ser139) antibody (CST, #9719) according to the manufacturer’s protocol.

### Protein Extraction and Western Blot

Total protein from HK-2 cells was extracted with RIPA buffer (Sigma, R0278), and protein from the kidneys of mice was extracted with a Minute^TM^ Total Protein Extraction Kit for Animal Cultured Cells/Tissues (Invent, SD-001) following standard protocols. Protein concentration was measured using a Pierce BCA Protein Assay kit (Thermo Fisher Scientific, 23227). Next, denatured proteins were separated in sodium dodecyl sulfate-polyacrylamide gels and then were electrically transferred onto polyvinylidene difluoride membranes (Millipore, IPVH00010). The membranes were blocked for 60 min in 5% fat-free milk dissolved in Tris-buffer saline with 0.1% Tween 20 (TBST). The blots were incubated with relevant primary antibodies overnight at 4°C as follows: γH2A.X (ser139) (Noves, NB100-74435, 1:1000), α-SMA (Abcam, ab32575, 1:2000), Collagen IV (Abcam, ab6586, 1:2000), Tubulin (ZSBio, TA-10, 1:5000) and GAPDH (Santa Cruz, sc-32233, 1:2000). After washing three times with TBST, the membranes were incubated with secondary HRP-conjugated secondary antibodies at a 1:1000 dilution for 1 h at room temperature. After five washes with TBST, the membranes were incubated in chemiluminescent substance (Millipore, WBKLS0100) for 5 min, and images were captured by a ImageQuant LAS 4000 Mini system (GE Healthcare). The density of each band was quantified by ImageJ (Media Cybernetics, Silver Spring, MD, United States).

### SA-β-gal Staining

Senescence-associated β-galactosidase (SA-β-gal) staining was performed using a senescence cell histochemical staining kit (Sigma-Aldrich, CS0030) according to the manufacturer’s instructions. For *in vitro* experiments, cells were evaluated under a light microscope, and SA-β-gal-positive cells were counted in at least ten fields. For *in vivo* experiments, frozen sections (4 μm thickness) of kidney tissues were used. At least fifteen fields were calculated under a light microscope, and the mean integrated optical density (IOD) of SA-β-gal expression was analyzed by Image-Pro Plus software (Media Cybernetics Co., Ltd).

### EdU Incorporation

DNA replication activity was analyzed in cells with an EdU staining kit (Thermo Fisher Scientific, C10337). Briefly, HK-2 cells were seeded on coverslips in 12-well plates as previously described. EdU (10 μM) was added to each well for 2 h until the cells were harvested 48 h after stimulation. Cells were collected and fixed with 4% paraformaldehyde for 10 min and then were permeabilized with 0.5% Triton X-100 for 10 min at room temperature. The cells were then stained with a Click-iT^®^ Plus reaction cocktail kit for 30 min at room temperature. Finally, images were obtained with a microscope (Nikon, Tokyo, Japan) and then were analyzed with Image J (Media Cybernetics, Silver Spring).

### Measurement of Cell Viability

HK-2 cells grown in 96-well plates were treated as previously described. Various concentrations of NMN (ranging from 0.03125 to 2 mM) were simultaneously added into the culture medium with H_2_O_2_ and while hypoxia stimuli was administered; treatment lasted 48 h. CellTiter-Fluor^TM^ Cell Viability Assay kit (Promega, Madison, WI, United States) was used to assess cell viability according to the manufacturer’s instructions. Following incubation of the cells with the substrate for 60 min at 37°C, fluorescence was measured using a Synergy H1 reader (excitation: 400 nm/emission: 505 nm). Viability of the treated cells was normalized against the control cells.

### Kidney Histopathological Analysis

To evaluate renal pathologic changes, kidney tissue samples were fixed overnight with 10% formalin in 0.01 mol/L phosphate buffer (pH 7.2) and then embedded in paraffin for histopathology analysis. The slide sections (3–4 μm thickness) were stained with hematoxylin-eosin (HE) according to standard procedures and examined under a light microscope. The examination of renal pathology was performed in a blinded fashion, and the pathologic assessment was performed on the basis of the percentage of tubules with necrosis, detachment, cast formation, dilation, or cell swelling.

### Sirius Red Staining

After deparaffinization and rehydration, paraffin sections were stained with Sirius red to evaluate collagen fibers according to manufacture’s instruction (Solarbio, G1470), and were calculated as a percentage of the total area. The images of Sirius red-stained sections were obtained with a digital microscope camera (Nikon, Tokyo, Japan), and quantitative evaluation was performed using Image-Pro Plus software (Media Cybernetics Co., Ltd).

### *In situ* TUNEL Assay

Apoptosis in the kidney tissues was detected in paraffin sections by *in situ* TUNEL assays that were performed according to a standard protocol (Beyotime Biotechnology, C1086). Ten to fifteen fields were selected randomly from each tissue section and the number of TUNEL-positive cells were determined per 400× field.

### Immunofluorescence Staining

Immunofluorescence staining of the kidney was performed on paraffin sections. After fixation and antigen retrieval, non-specific binding was blocked with 3% BSA. Kidney sections were then incubated with the following primary antibodies: rabbit antibody to α-SMA (Abcam, ab32575, 1:200), rabbit antibody to Ki-67 (Abcam, ab66155, 1:500), and mouse antibody to p-H3 (ser10) (Abcam, ab14955, 1:1000). The slides were then exposed to FITC or Cy3-labeled secondary antibodies (Jackson ImmunoResearch) and were mounted with medium containing DAPI. The percentage of α-SMA-positive area to cortex and outer medulla section were calculated, respectively, using Image-Pro Plus software (Media Cybernetics Co., Ltd). For cell cycle analysis, results are expressed as the number of Ki-67 or p-H3 positive tubular cells per high-power field.

### RNA Isolation and RT-PCR Analysis

Kidney tissues were collected in RNase-free tubes, and total RNA was extracted using TRIzol reagent (Invitrogen) according to the manufacturer’s instructions. For cDNA synthesis, reverse transcription was performed from 2 μg of total RNA using a FastKing RT Kit (Tiangen, KR116). The mRNA expression levels of IL-6, IL-8, TGF-β1, and β-actin were determined using SuperReal PreMix Plus (SYBR Green) (FP205, Tiangen) based on the manufacturer’s instructions. The sequences of the primers used are shown in Table 1. The PCR system consisted of SYBR Green Mix, forward and reverse primers, cDNA, and deionized RNase-free water. PCR was initially denatured at 95°C for 30 s followed by 95°C for 10 s and 65°C for 30 s for 40 cycles and then 81 cycles at 55–95°C for 10 s for melting curve analysis. The comparative gene expression was calculated by the 2^–ΔΔCt^ method as described previously.

**TABLE 1:** Primers used for real-time PCR.

**Genes**	**Forward primers (5′-3′)**	**Reverse primers (5′-3′)**
IL-6	CTGCAAGAGACTTCCATCCAG	AGTGGTATAGACAGGTCTGTTGG
IL-8	TCGAGACCATTTACTGCAACAG	CATTGCCGGTGGAAATTCCTT
TGF-β1	CTCCCGTGGCTTCTAGTGC	GCCTTAGTTTGGACAGGATCTG
β-actin	CAGCTGAGAGGGAAATCGTG	CGTTGCCAATAGTGATGACC

*PCR, polymerase chain reaction; IL-6, interleukin-6; IL-8, interleukin-8; TGF-β1, transforming growth factor β1.*

### Statistical Analysis

GraphPad Prism 6.0 was used. Data from repeated experiments were analyzed and are shown as the mean ± SD. A two-tailed unpaired *t*-test was applied for comparisons between two groups. Differences at the *P* < 0.05 level were considered statistically significant.

## Results

### Hydrogen Peroxide and Hypoxia Stimulation Resulted in DNA Damage, G2/M Arrest, and Senescence in HK-2 Cells

**FIGURE 1:**
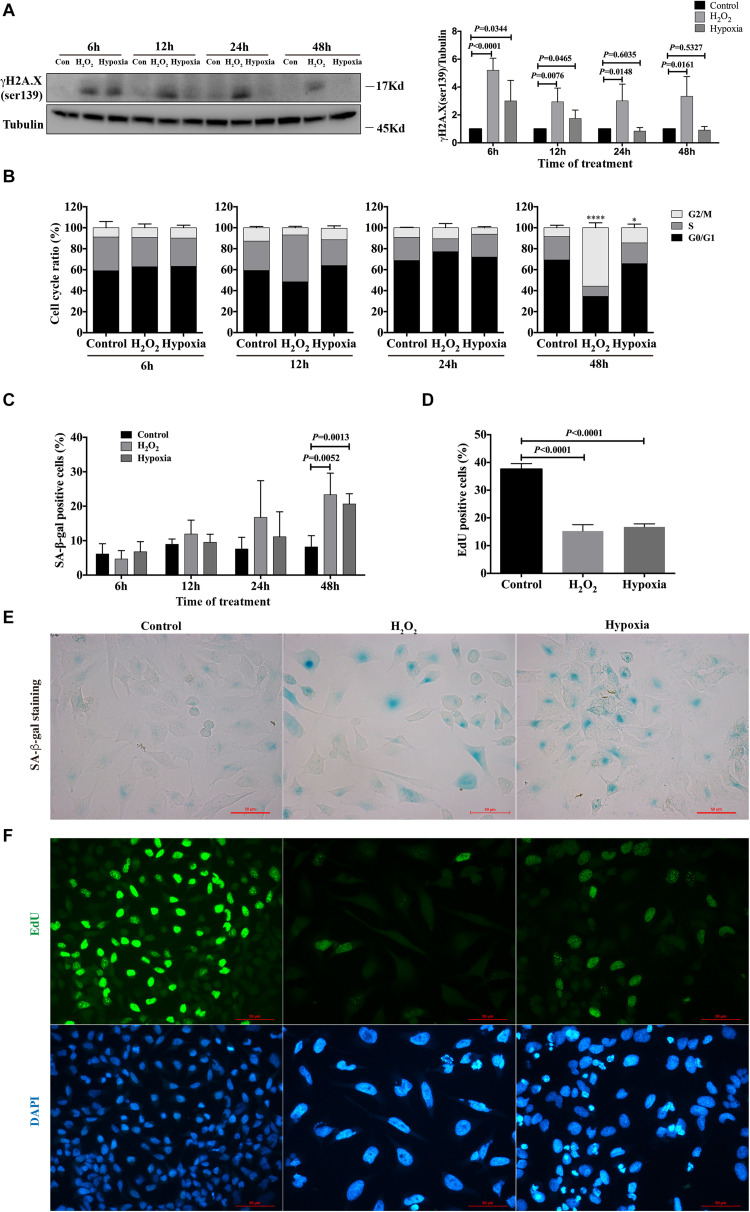
Hydrogen peroxide and hypoxia resulted in DNA damage, G2/M arrest and senescence in HK-2 cells. **(A)** Representative western blots analysis of γH2A.X (ser139), and bar graphs showed the fold changes compared to control group at different time points. **(B)** Flow cytometry analysis of cell cycle. *P < 0.05, ****P < 0.0001, compared with control group. **(C)** SA-β-gal staining analysis at different time points. **(D)** EdU incorporation analysis at 48 h. **(E)** Representative SA-β-gal staining at 48 h, Scale bars, 50 μm. **(F)** Representative EdU incorporation images at 48 h showed decreased proliferation rate and larger cell size in H_2_O_2_ and hypoxia groups, Scale bars, 50 μm. *n* = 3–5/group. Data are means ± SD. SA-β-gal, Senescence Associated β-Galactosidase; EdU, 5-Ethynyl-2′-deoxyuridine.

To evaluate the phenotype of injury caused by stimuli in renal tubular cells *in vitro*, HK-2 cells were treated with hydrogen peroxide (H_2_O_2_) and subjected to hypoxia (1% O2). After 6, 12, 24 and 48 h, the cells were harvested, and the degree of DNA damage, cell cycle distribution and senescence were examined. By western blot analysis, we found that the expression of γH2A.X (ser139), a DNA damage marker (Rogakou et al., 1998; Sharma et al., 2012), was significantly enhanced in both H_2_O_2_- and hypoxia-treated groups at 6 h, and its expression were sustained for the duration of the study for the H_2_O_2_-treated group (Figure 1A). For the hypoxia-treated group, the enhanced expression of γH2A.X (ser139) was rarely detected at 24 and 48 h (Figure 1A). Cell cycle analysis showed that H_2_O_2_ and hypoxia treatment did not cause significant changes in cell cycle distribution at 6, 12, and 24 h. However, the percentage of cells in G2/M increased significantly in the H_2_O_2_- and hypoxia-treated groups at 48 h compared with the control group (8.450% ± 2.350%), being 55.72% ± 4.682% (*P* < 0.0001) and 14.42% ± 3.485% (*P* = 0.0131), respectively (Figure 1B). Meanwhile, the percentages of senescent cells in both the H_2_O_2_- and hypoxia-treated groups increased from 8.185% ± 1.629% to 23.33% ± 3.140% and 20.65% ± 1.491%, respectively (Figures 1C,E), as reflected by SA-β-gal staining. EdU incorporation analysis showed significantly decreased proliferation rates of HK-2 cells in the H_2_O_2_ and hypoxia groups at 48 h (Figures 1D,F). These results suggest that H_2_O_2_ and hypoxia stimuli could induce DNA damage in HK-2 cells and might thereby further result in G2/M arrest or senescence.

### NMN Increased Cell Viability and Attenuated DNA Damage, Senescence and Collagen Production in HK-2 Cells After Hydrogen Peroxide and Hypoxia Stimulation

**FIGURE 2:**
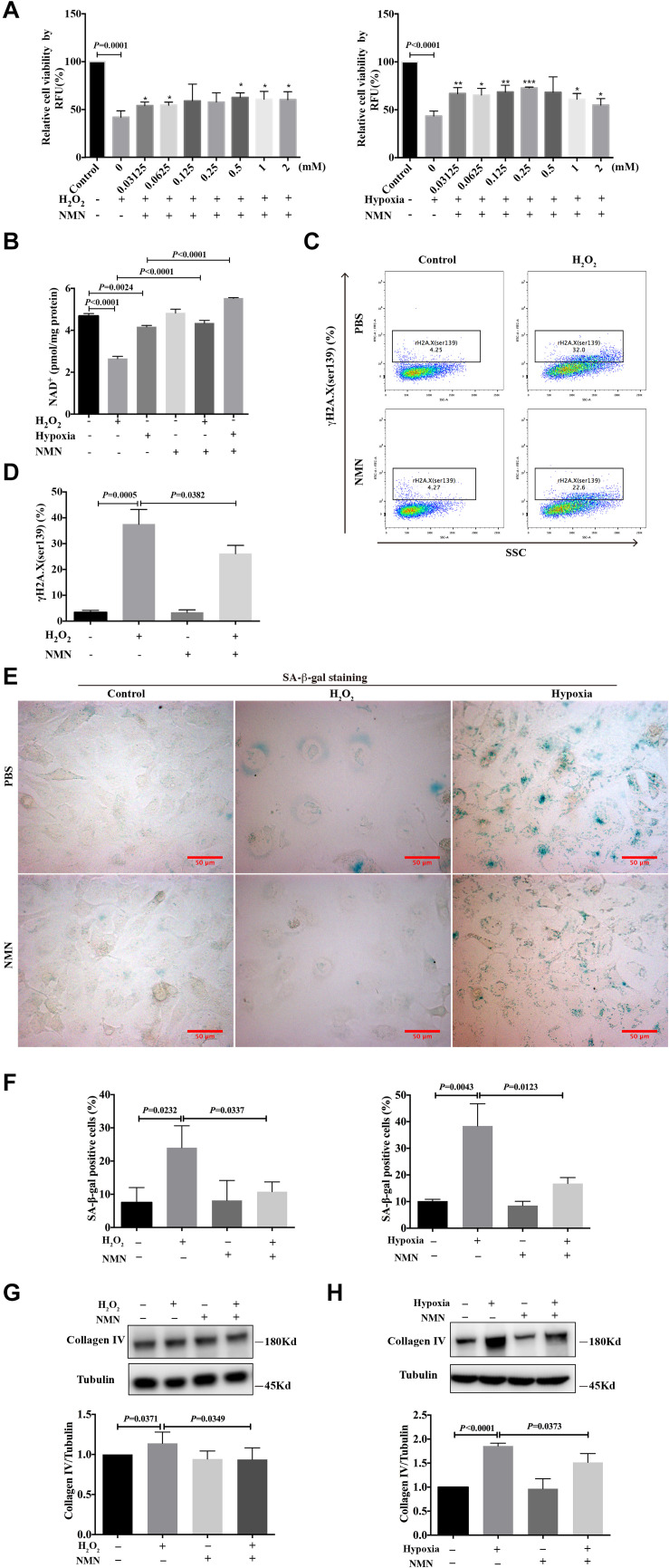
Nicotinamide mononucleotide (NMN) attenuated hydrogen peroxide and hypoxia induced injuries and senescence in HK-2 cells. **(A)** Cell viability percentage of HK-2 cells. *P < 0.05, **P < 0.01, ***P < 0.001 compared with H_2_O_2_ or hypoxia group. **(B)** Quantification of NAD^+^ levels in HK-2 cells. **(C,D)** Flow cytometry analysis of γH2A.X (ser139) in H_2_O_2_ group, and bar graph showed decreased DNA damage in NMN-treated group. **(E)** Representative SA-β-gal staining images, Scale bars, 50 μm. **(F)** Bar graphs showing significantly decreased percentage of SA-β-gal positive cells in NMN-treated groups at a dose of 1 mM. **(G,H)** Western blots analysis showed reduction of collagen IV in NMN-treated groups, compared with H_2_O_2_ or hypoxia group. *n* = 3–5/group. Data are means ± SD. NMN, Nicotinamide Mononucleotide.

To investigate the effect of nicotinamide mononucleotide (NMN) on the injury phenotypes induced by H_2_O_2_ and hypoxia, HK-2 cells were simultaneously incubated for 48 h with various doses of NMN, during H_2_O_2_ and hypoxia exposure. CellTiter-Fluor^TM^ cell viability assay was performed to examine cell viability. As shown in Figure 2A, NMN administration significantly enhanced the decreased cell viability caused by H_2_O_2_ and hypoxia stimulation in a dose-dependent manner starting at the lowest dose tested. Considering the improved cell viability and convenience of calculation, NMN at a dose of 1 mM was selected for the following *in vitro* studies. NMN could significantly restored decreased HK-2 cellular NAD^+^ levels caused by H_2_O_2_ and hypoxia insult (Figure 2B). To explore the protective effect of NMN on DNA damage, the expression level of γH2A.X (ser139) was detected by flow cytometry. We found that the percentage of DNA-damaged cells was markedly decreased from 32.0% to 22.6% (*P* = 0.0382) by NMN administration in the H_2_O_2_-treated group at 48 h (Figures 2C,D). In addition, NMN administration resulted in a decreased percentage of SA-β-gal-positive cells in the H_2_O_2_ and hypoxia groups (Figures 2E,F), indicating the effective antiaging activity of NMN *in vitro*. Furthermore, collagen IV protein production in H_2_O_2_- and hypoxia-treated HK-2 cells was increased, but it could be suppressed by NMN administration, as determined by western blot analysis (Figures 2G,H). Similar protective effect of NMN was found in HK-2 cells subjected to hypoxia followed by oxygenation (Supplementary Figure 1). These *in vitro* data suggest the protective effect of NMN on tubular cell injury.

### NMN Administration Before Ischemia and During the Acute Phase Attenuated Renal Tubular DNA Damage and Cellular Injury in uIRI Mice

**FIGURE 3:**
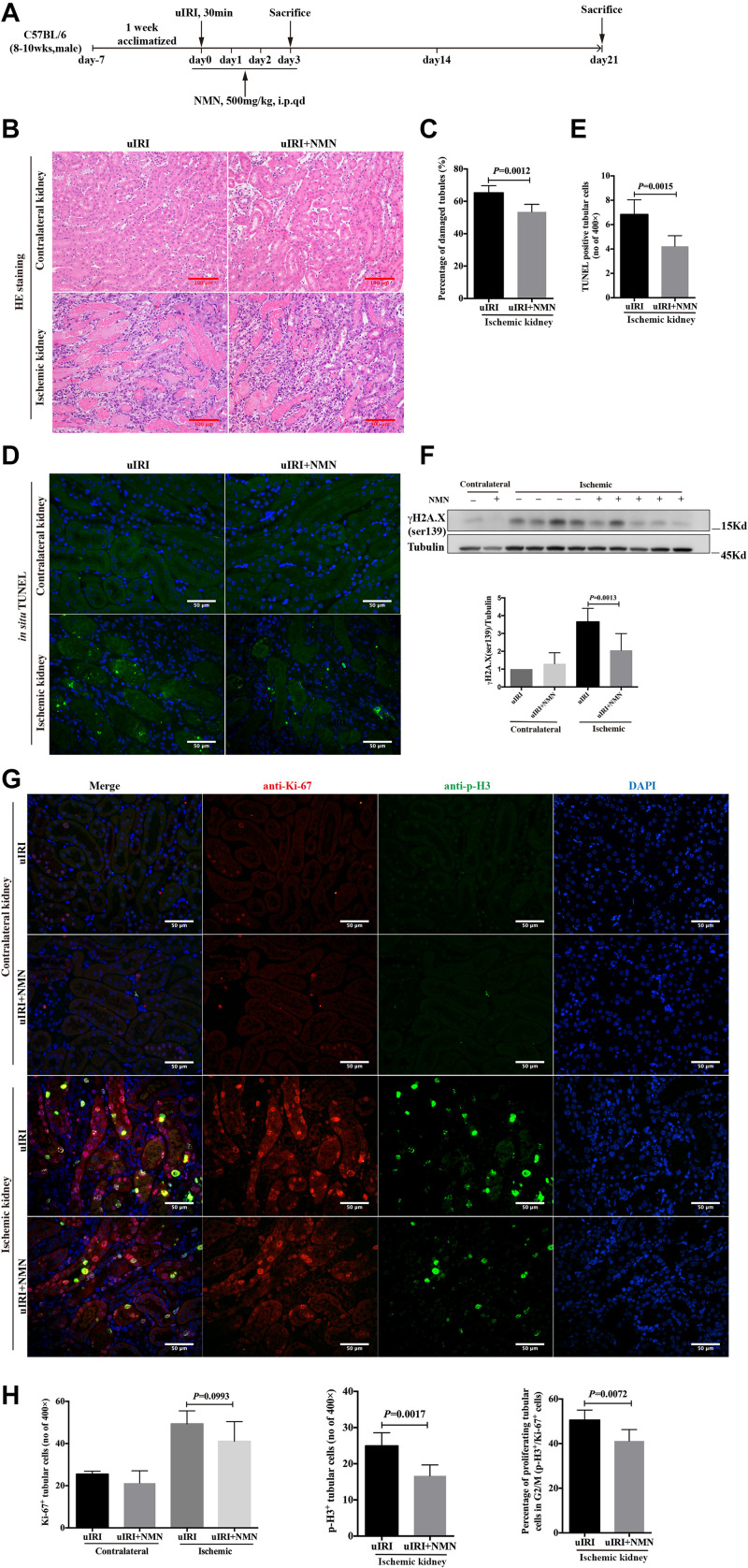
Nicotinamide mononucleotide (NMN) administration before ischemia and during acute phase attenuated tubular DNA damage and cellular injury in uIRI mice at day 3 after surgery. **(A)** Schematic protocol of the experiments. **(B)** Representative images of HE (hematoxylin-eosin) staining and histologic analysis of tubular damage. Scale bars, 100 μm. **(C)** Quantification of damaged tubules of ischemic kidneys in **(B)**. **(D)** Representative images of *in situ* TUNEL assay. Scale bars, 50 μm. **(E)** Quantification of apopotic tubular cells in ischemic kidneys in **(D)**. **(F)** Western blots analysis of γH2A.X (ser139) showed decreased DNA damage in ischemic kidneys of NMN-treated mice compared with ischemic kidneys of PBS-treated mice. **(G)** Representative images of co-immunostaining of anti-Ki-67 and anti-p-H3 in kidney tissues. Scale bars, 50 μm. **(H)** Bar graphs showed no changes in number of Ki-67 positives tubular cells in NMN-treated mice, but a markedly reduction of tubular cells in G2/M phase compared with PBS-treated mice. *n* = 6–10/group. Data are means ± SD.

To confirm the protective effects of NMN *in vivo*, we established an uIRI mouse model, and NMN or PBS was injected intraperitoneally 20 min before surgery as well as on days 1, 2, and 3 after surgery (totaling 4 consecutive days of injection) (Figure 3A). The mice were sacrificed 4 h after the last injection at day 3 after surgery, and both the ischemic left and healthy right kidneys were taken. Histological study showed intact structure in healthy right kidneys (Figure 3B). Significant renal tubular injury were seen in PBS-treated ischemic kidney, including severe dilation of the proximal tubules, cast formation, and massive detachment and necrosis of the tubular epithelium, while NMN-treated ischemic kidney showed significantly decreased tubular injury (Figures 3B,C). We further conducted an *in situ* TUNEL apoptosis assay and found an increase in tubular apoptosis after injury in PBS-treated mice, while NMN administration substantially reduced tubular apoptosis (*P* = 0.0015, Figures 3D,E). In addition, uIRI mice had elevated levels of DNA damage at day 3 after surgery, as determined by western blot analysis of γH2A.X (ser139), and NMN administration significantly decreased the upregulation of DNA damage (Figure 3F). This suggests that NMN has a protective effect against DNA damage *in vivo*, which is consistent with the *in vitro* results. To analyze the proliferation and cell cycle distribution of proximal tubular epithelial cells, immunohistochemistry co-staining of Ki-67 and phosphorylation of histone H3 at ser10 (p-H3) were performed. The uIRI mice had an increase in tubular cell proliferation (Ki-67 positive) at day 3 after injury, and NMN administration did not cause obvious changes in the number of proliferating tubular cells. There was also an increase in the number of tubular cells in G2/M phase (p-H3 positive) after injury, and the percentage of tubular cells in G2/M phase (p-H3 positive) among all proliferating tubular cells decreased significantly in NMN-treated uIRI mice (from 50.53% ± 1.828% to 41.19% ± 2.093%, *P* = 0.0072) (Figures 3G,H). This suggested that NMN administration before ischemia and during the acute phase could attenuate renal tubular DNA damage and cellular injury in uIRI mice.

### NMN Administration Before Ischemia and During the Acute Phase Attenuated Renal Tubular Senescence, Chronic Inflammation and Fibrosis in uIRI Mice

**FIGURE 4:**
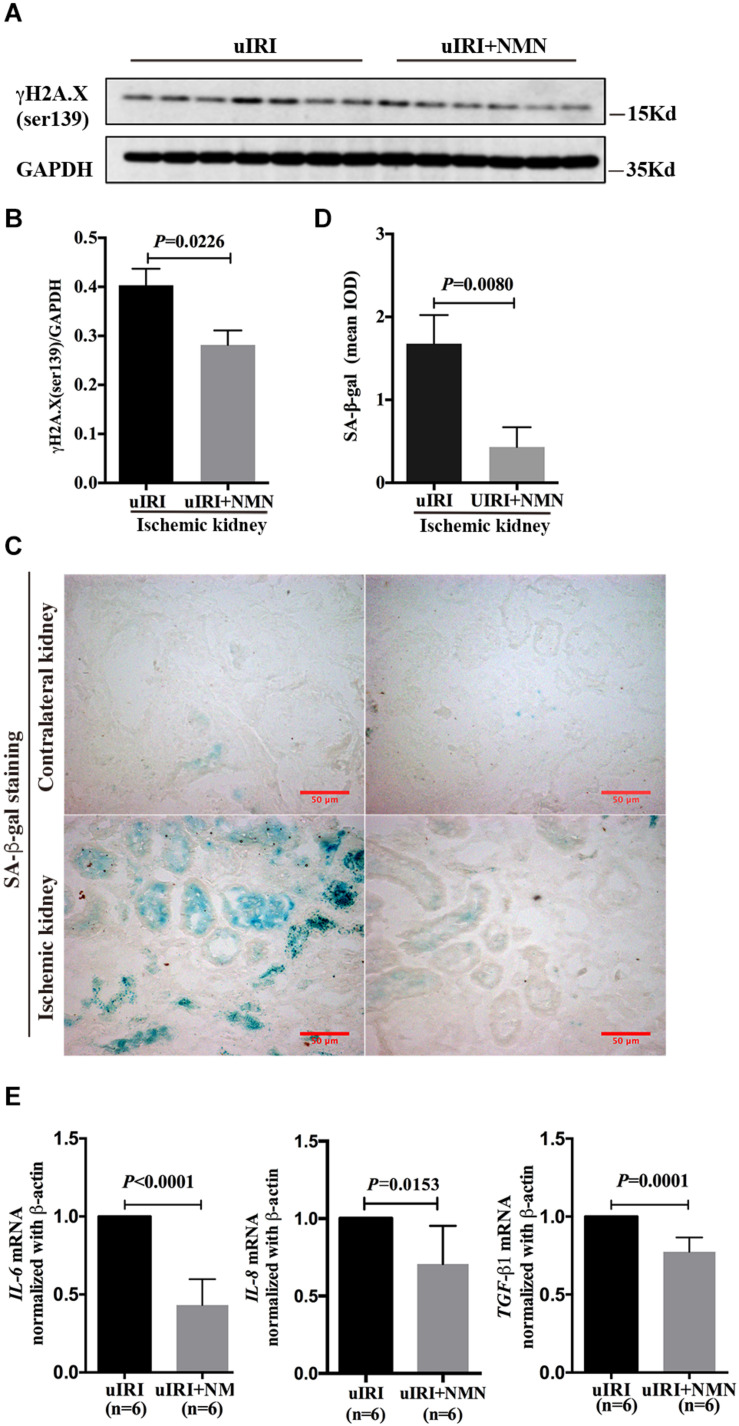
Nicotinamide mononucleotide (NMN) administration before ischemia and during acute phase attenuated tubular senescence in uIRI mice at day 21 after surgery. **(A)** Representative western blots analysis of γH2A.X (ser139). **(B)** Bar graph showed reduction of DNA damage in NMN-treated group. **(C,D)** SA-β-gal staining analysis showed decreased senescent tubular cells in NMN-treated uIRI mice. Scale bars, 50 μm. **(E)** Relative expression of IL-6, IL-8, and TGF-β1 mRNAs after normalization with β-actin. *n* = 6–7/group. Data are means ± SD.

**FIGURE 5:**
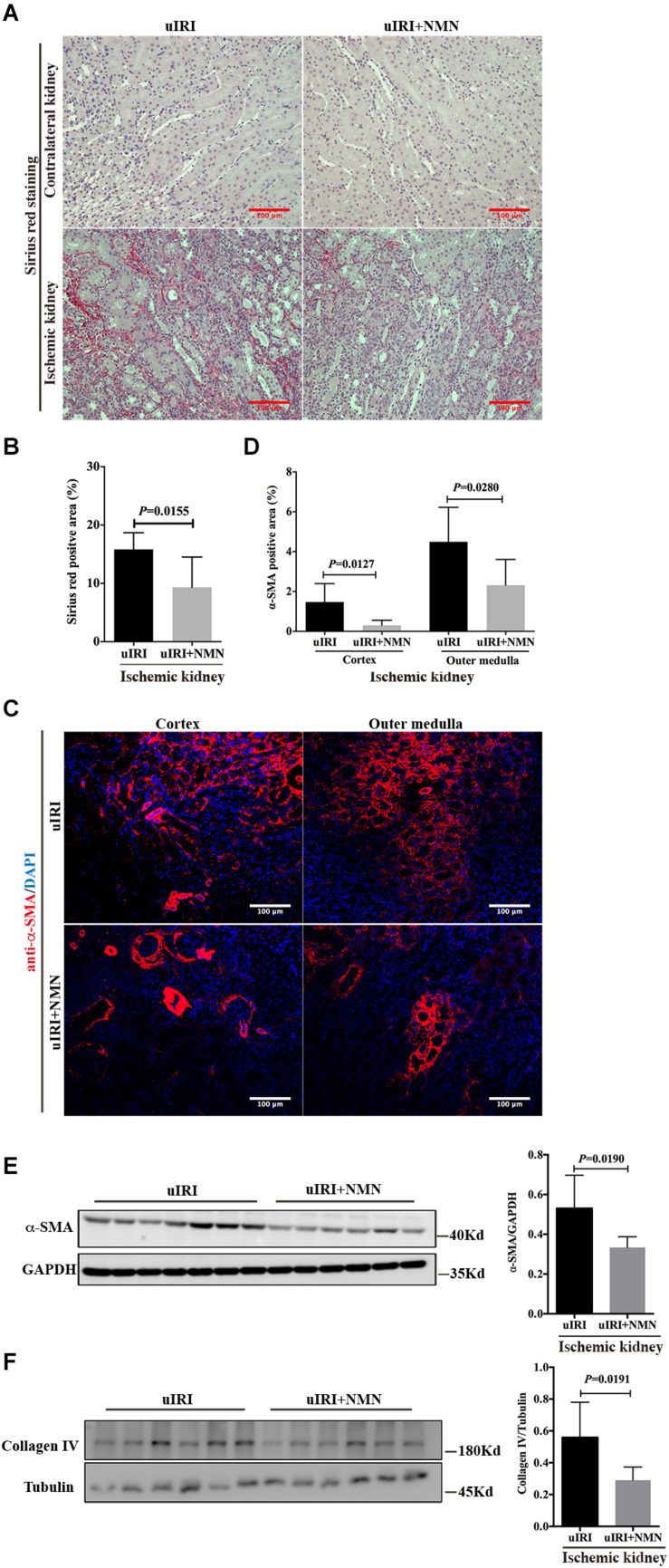
Nicotinamide mononucleotide (NMN) administration before ischemia and during acute phase attenuated uIRI induced interstitial fibrosis at day 21 after surgery. **(A,B)** Representative sirius red staining images and semiquantitative analysis of the percentage of positive sirius red staining area. Scale bars, 100 μm. **(C,D)** Representative images of alpha-smooth muscle actin (α-SMA) immunofluorescence staining in cortex and outer medulla and semiquantitative analysis of percentage of positive area. Scale bars, 100 μm. **(E)** Representative western blots and semiquantitative analysis of α-SMA. **(F)** Representative western blots and semiquantitative analysis of Collagen IV (Col IV). *n* = 6–7/group. Data are means ± SD.

To explore the effect of NMN on chronic kidney changes in uIRI mice, kidneys of uIRI mice were treated with PBS or NMN as described above, and then they were collected at day 21 after surgery. We found that tubular DNA damage was sustained at day 21 after surgery and could be significantly suppressed by NMN administration, as reflected by western blot analysis of γH2A.X (ser139) (Figures 4A,B). Furthermore, the tubular cells of uIRI mice showed strong positive SA-β-gal staining, indicating that the cells were senescent, which could also be significantly reduced by NMN treatment (Figures 4C,D). Senescent tubular cells are thought to secrete cytokines and inflammatory factors, which are indicative of the senescence-associated secretory phenotype (SASP). SASP composition varies depending on cell and tissue of origin and the triggers involved, but IL-6 and IL-8 are a highly conserved part of the SASP and have an important role in propagating senescence and regulating its accompanying inflammatory phenotype. As shown by the qRT-PCR data (Figure 4E), the mRNA levels of *IL-6*, *IL-8*, and *TGF-β1* were decreased in NMN-treated uIRI mice. These data indicated an inhibitory effect of NMN on uIRI-induced tubular premature senescence. Sirius red staining revealed a high level of collagen deposition in PBS-treated uIRI mice (Figure 5A), which was alleviated significantly in NMN-treated mice (*P* = 0.0155) (Figure 5B). Immunostaining of α-SMA together with western blot analysis of α-SMA and collagen IV confirmed the significantly decreased collagen deposition in NMN-treated uIRI mice (Figures 5C–F). These results indicate that fibrotic changes in the kidneys of uIRI mice could be alleviated by the administration of NMN before ischemia and during the acute phase.

### NMN Administration During the Recovery Phase Attenuated the uIRI-Induced DNA Damage, Senescence and Fibrosis Phenotype

**FIGURE 6:**
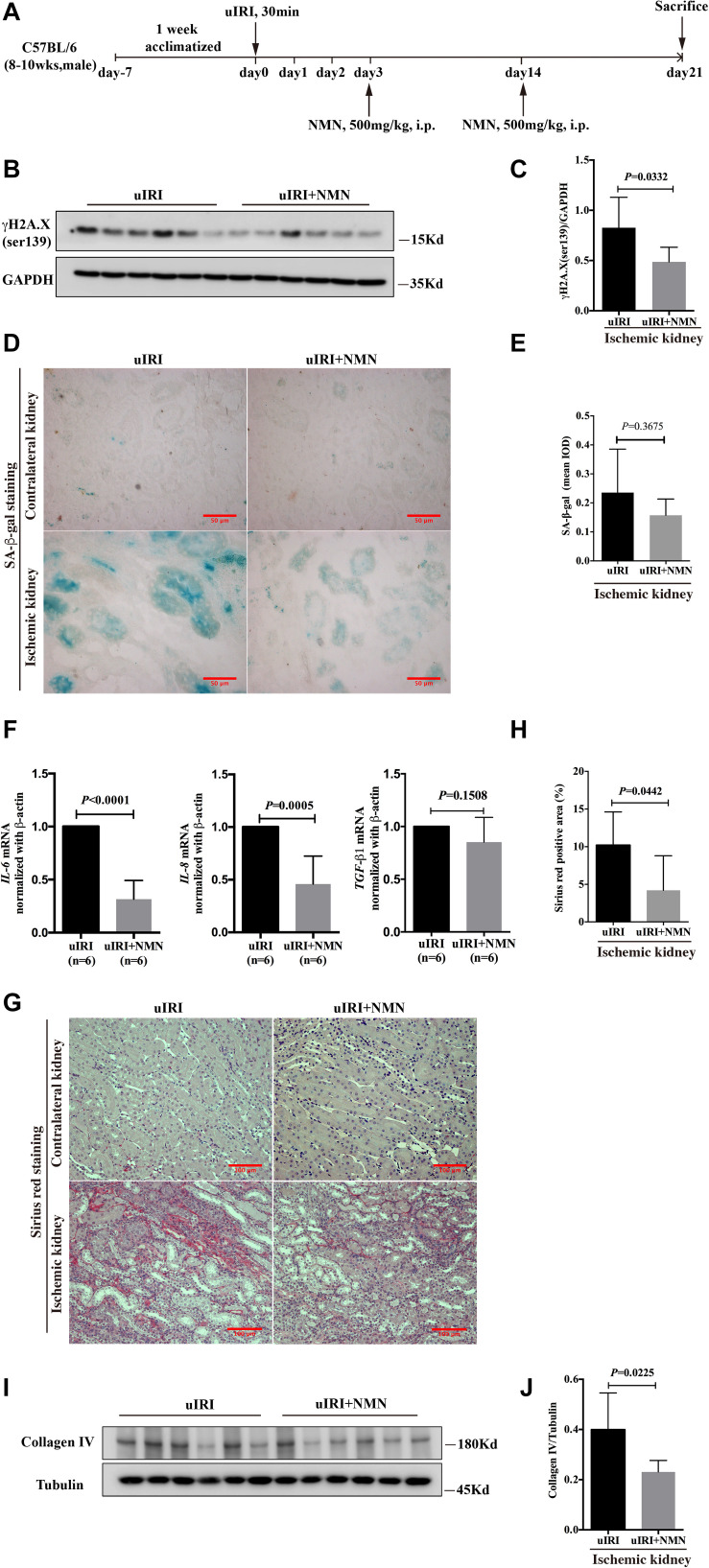
Nicotinamide mononucleotide (NMN) administration at recovery phase attenuated uIRI induced DNA damage, inflammation and fibrosis at day 21 after surgery. **(A)** Schematic protocol of the experiments. **(B,C)** Representative western blots analysis of γH2A.X (ser139), and bar graph showed reduction of DNA damage in NMN-treated group. **(D)** Representative images of SA-β-gal staining in both groups. Scale bars, 50 μm. **(E)** Quantification of expression of SA-β-gal in **(D)**. **(F)** Relative expression of IL-6, IL-8, and TGF-β1 mRNA after normalization with β-actin. **(G,H)** Representative sirius red staining images and semiquantitative analysis of the percentage of positive area. Scale bars, 100 μm. **(I,J)** Representative western blots and semiquantitative analysis of Collagen IV (Col IV), *n* = 6/group. Data are means ± SD.

Considering that the use of prophylactic medication is not practical in clinical patients, we evaluated the renal fibrosis inhibitory effect of NMN administration during the recovery phase of IRI. Mice that underwent uIRI surgery were injected with NMN intraperitoneally on days 3 and 14 after surgery and were sacrificed on day 21 (Figure 6A). NMN administration significantly improved NAD^+^ levels in the ischemic kidney (Supplementary Figure 2). And we found that the expression level of γH2A.X (ser139) was significantly decreased in NMN-treated mice at day 21 (Figures 6B,C), which is consistent with the observations of NMN administration before ischemia and during the acute phase. NMN administration also alleviated SA-β-gal positive staining in renal tubular cells in NMN-treated uIRI mice but did not reach statistical significance (Figures 6D,E). The chronic interstitial inflammation reflected by the mRNA levels of *IL-6* and *IL-8* was reduced significantly in NMN-treated uIRI mice (Figure 6F). *TGF-β1* mRNA level also decreased, but didn’t reach statistical significance. These observations suggest that NMN could inhibit DNA damage and might reduce senescence of renal tubular cells even during the recovery phase of uIRI. Sirius red staining revealed decreased collagen deposition in NMN-treated uIRI mice (Figures 6G,H). Additionally, the expression level of collagen IV was decreased significantly in the NMN-treated group compared with the PBS group (Figures 6I,J). These results indicate that fibrotic changes in the kidneys of uIRI mice could be alleviated by NMN administration even during the recovery phase.

## Discussion

Considering the high incidence of AKI and the high proportion of patients progressing to chronic kidney disease (CKD) and end stage renal disease (ESRD) (Yang, 2019), there is an urgent need to identify effective treatments for fibrosis after AKI. To our knowledge, this is the first report that NMN, an NAD^+^ precursor, could attenuate long-term fibrotic responses following experimental ischemic AKI, whether administered in advance or during the recovery phase. Our results also indicated that the anti-fibrotic effect of NMN might be achieved by reducing renal tubular DNA damage, and thus decreasing tubular senescence and senescence associated inflammation.

DNA damage is a feature of many forms of kidney injury, and it activates specific cell signaling cascades for DNA repair, cell cycle arrest, senescence, and cell death (Tsuruya et al., 2003; Yang et al., 2010; Zhu et al., 2015). Thus, targeting DNA damage and repair might be an effective strategy for protecting kidneys in AKI. NAD^+^ participates in DNA repair by serving as a substrate for poly-ADP-ribose polymerase (PARP) and sirtuins and by providing adenylate to DNA ligase IV, a key enzyme for DNA double-strand break (DSB) repair (de Murcia and Menissier de Murcia, 1994; de Murcia et al., 1994; Kim et al., 2004; De Vos et al., 2012; Fouquerel and Sobol, 2014; Fang et al., 2016; Chen and Yu, 2019). Thus, boosting NAD^+^ levels was predicted to have beneficial effects in DNA damage repair. Researchers have found that NMN could repair damaged DNA in the livers of old mice (Li et al., 2017) and that it could maintain telomere length, dampen the DNA damage response and rescue liver fibrosis in the livers of telomerase knockout mice (Amano et al., 2019). However, no kidney-related studies have been performed to date. In this study, we observed that stress-induced renal tubular DNA damage occurred upon acute insult and was sustained when subsequent chronic changes (senescence and fibrosis) occurred. And NMN administration both *in vivo* and *in vitro* showed reduced DNA damage phenotype. This gives evidence that boosting NAD^+^ might help to improve DNA repair capacity of the injured tissues, but direct correlation needs to be further studied. A recent encouraging study by Canto et al. showed that dihydronicotinamide riboside (NRH, a reduced form of NR) had unprecedented ability to increase NAD^+^ levels and could counteract cisplatin-induced kidney cellular damage and renal function decline (Giroud-Gerbetant et al., 2019). Therefore, it’s promising to evaluate the ability of NRH in preventing or treating renal fibrosis of various etiologies in the future.

Repeated or prolonged damage (ischemia or toxic injury) of tubular epithelial cells leads to senescence and maladaptive repair (Yang et al., 2010). Senescent cells often acquire a senescence-associated secretory phenotype (SASP), which is characterized by the expression and secretion of profibrotic and proinflammatory factors and are thought to be an important driver of fibrosis (Coppe et al., 2010; de Keizer, 2017). Recent evidence suggests that tubular senescence may play a key role in CKD progression (Valentijn et al., 2018). Senescent cell manipulation and depletion might represent novel therapies for treating AKI. In this study, we found that NMN markedly attenuated tubular senescence and blocked the accumulation of extracellular matrix (ECM) components both *in vitro* and *in vivo*. We also observed a reduction in the mRNA levels of *IL-6* and *IL-8* following NMN administration in the uIRI model, which might be due to the amelioration of senescence in tubular cells.

Another important finding of this study is that the protective effects of NMN on DNA damage inhibition, antisenescence and antifibrosis could be observed even when NMN was administered during the recovery phase of the ischemic AKI model. Although some studies have suggested the protective role of NAD^+^ precursors for renal function in various etiologies of experimental AKI models (Tran et al., 2016; Guan et al., 2017), in these studies, NMN was administered in advance. The use of prophylactic medication is not practical in many clinical conditions. Our study indicated a wide therapeutic time window of NMN in an ischemic AKI mouse model. Recently, several clinical trials have been carried out to determine the pharmacokinetics (US National Library of Medicine, 2014, 2015, 2016c) and bioavailability (US National Library of Medicine, 2017) of some NAD^+^ precursors, such as NMN, NR, and nicotinamide (NAM) (Tsubota, 2016). The efficacy of NR supplementation in obesity and insulin sensitivity is being tested (US National Library of Medicine, 2016a, b), and no adverse effects have been reported. Therefore, although preliminary, our results suggest that NMN administration might be an effective strategy for preventing or treating kidney fibrosis after AKI. For continued development and clinical translation, further elucidation of the mechanisms behind the therapeutic effects of NMN is needed. Preclinical studies in kidney and other organs suggested that augmentation of NAD^+^ not only enable efficient ATP production via fatty acid oxidation (FAO) but also broad cell-regulatory signaling networks that protect oxidative metabolism and mitochondrial health by acting as PARP1 and CD38 inhibitor (Li et al., 2017), sirtuins activator (Guan et al., 2017), mitochondrial fission inhibitor (Klimova et al., 2019; Lynch et al., 2019). Efficient FAO might also prevents toxic effect of accumulated lipids (Tran et al., 2016). Phosphorylation of NAD^+^ to NADP^+^ may potentiate defense against oxidant stress by promoting the reduction of glutathione and through the vasodilator nitric oxide (Ratliff et al., 2016). In order to understand the complex NMN-related network changes, high-throughput methods are indispensable, such as transcriptome, proteome, and metabolomics. The use of tracers to track the metabolic changes of NMN in circulation and different organs is helpful to study the direct mechanism and the feasibility of its application in different diseases.

Collectively, our results suggest the DNA damage inhibition, antiaging and anti-inflammatory effects of NMN in kidneys, and NMN administration might be an effective strategy for preventing or treating kidney fibrosis after AKI.

## Data Availability Statement

The raw data supporting the conclusions of this article will be made available by the authors, without undue reservation.

## Ethics Statement

The animal study was reviewed and approved by the Institutional Animal Care and Use Committee of Peking University First Hospital.

## Author Contributions

YJ collected the data, analyzed the data, interpreted the results, and drafted the article. LY conceived, designed, and organized the study. LY and ZX interpreted the results and revised the manuscript. XK, LT, YR, LQ, and JT contributed to collecting samples. GL and SW contributed to pathologic analysis of kidney tissue. All authors contributed to the article and approved the submitted version.

## Conflict of Interest

The authors declare that the research was conducted in the absence of any commercial or financial relationships that could be construed as a potential conflict of interest.

## References

[B1] Amano H. Chaudhury A. Rodriguez-Aguayo C. Lu L. Akhanov V. Catic A. (2019). Telomere dysfunction induces sirtuin repression that drives telomere-dependent disease. *Cell. Metab.* 29 1274–1290. 10.1016/j.cmet.2019.03.001 30930169 PMC6657508

[B2] Bonventre J. V. Yang L. (2011). Cellular pathophysiology of ischemic acute kidney injury. *J. Clin. Invest.* 121 4210–4221. 10.1172/JCI45161 22045571 PMC3204829

[B3] Canaud G. Brooks C. R. Kishi S. Taguchi K. Nishimura K. Magassa S. (2019). Cyclin G1 and TASCC regulate kidney epithelial cell G2-M arrest and fibrotic maladaptive repair. *Sci. Transl. Med.* 11:eaav4754. 10.1126/scitranslmed.aav4754 30674655 PMC6527117

[B4] Chen S. H. Yu X. (2019). Human DNA ligase IV is able to use NAD+ as an alternative adenylation donor for DNA ends ligation. *Nucleic Acids Res.* 47 1321–1334. 10.1093/nar/gky1202 30496552 PMC6379666

[B5] Coppe J. P. Desprez P. Y. Krtolica A. Campisi J. (2010). The senescence-associated secretory phenotype: the dark side of tumor suppression. *Annu. Rev. Pathol.* 5 99–118. 10.1146/annurev-pathol-121808-102144 20078217 PMC4166495

[B6] de Keizer P. L. (2017). The fountain of youth by targeting senescent cells? *Trends Mol. Med.* 23 6–17. 10.1016/j.molmed.2016.11.006 28041565

[B7] de Murcia G. Menissier de Murcia J. (1994). Poly(ADP-ribose) polymerase: a molecular nick-sensor. *Trends Biochem. Sci.* 19 172–176. 10.1016/0968-0004(94)90280-1 8016868

[B8] de Murcia G. Schreiber V. Molinete M. Saulier B. Poch O. Masson M. (1994). Structure and function of poly(ADP-ribose) polymerase. *Mol. Cell. Biochem.* 138 15–24. 10.1007/bf00928438 7898458

[B9] De Vos M. Schreiber V. Dantzer F. (2012). The diverse roles and clinical relevance of PARPs in DNA damage repair: current state of the art. *Biochem. Pharmacol.* 84 137–146. 10.1016/j.bcp.2012.03.018 22469522

[B10] Fang E. F. Kassahun H. Croteau D. L. Scheibye-Knudsen M. Marosi K. Lu H. (2016). NAD(+) Replenishment improves lifespan and healthspan in ataxia telangiectasia models via mitophagy and DNA repair. *Cell. Metab.* 24 566–581. 10.1016/j.cmet.2016.09.004 27732836 PMC5777858

[B11] Ferenbach D. A. Bonventre J. V. (2015). Mechanisms of maladaptive repair after AKI leading to accelerated kidney ageing and CKD. *Nat. Rev. Nephrol.* 11 264–276. 10.1038/nrneph.2015.3 25643664 PMC4412815

[B12] Fouquerel E. Sobol R. W. (2014). ARTD1 (PARP1) activation and NAD(+) in DNA repair and cell death. *DNA Repair (Amst)* 23 27–32. 10.1016/j.dnarep.2014.09.004 25283336 PMC4252787

[B13] Giroud-Gerbetant J. Joffraud M. Giner M. P. Cercillieux A. Bartova S. Makarov M. V. (2019). A reduced form of nicotinamide riboside defines a new path for NAD(+) biosynthesis and acts as an orally bioavailable NAD(+) precursor. *Mol. Metab.* 30 192–202. 10.1016/j.molmet.2019.09.013 31767171 PMC6807296

[B14] Guan Y. Wang S. R. Huang X. Z. Xie Q. H. Xu Y. Y. Shang D. (2017). Nicotinamide mononucleotide, an NAD(+) precursor, rescues age-associated susceptibility to AKI in a sirtuin 1-dependent manner. *J. Am. Soc. Nephrol.* 28 2337–2352. 10.1681/ASN.2016040385 28246130 PMC5533221

[B15] Kim M. Y. Mauro S. Gevry N. Lis J. T. Kraus W. L. (2004). NAD+-dependent modulation of chromatin structure and transcription by nucleosome binding properties of PARP-1. *Cell* 119 803–814. 10.1016/j.cell.2004.11.002 15607977

[B16] Kishi S. Brooks C. R. Taguchi K. Ichimura T. Mori Y. Akinfolarin A. (2019). Proximal tubule ATR regulates DNA repair to prevent maladaptive renal injury responses. *J. Clin. Invest.* 129 4797–4816. 10.1172/JCI122313 31589169 PMC6819104

[B17] Klimova N. Long A. Kristian T. (2019). Nicotinamide mononucleotide alters mitochondrial dynamics by SIRT3-dependent mechanism in male mice. *J. Neurosci. Res.* 97 975–990. 10.1002/jnr.24397 30801823 PMC6565489

[B18] Lee H. T. Kim M. Jan M. Emala C. W. (2006). Anti-inflammatory and antinecrotic effects of the volatile anesthetic sevoflurane in kidney proximal tubule cells. *Am. J. Physiol. Renal Physiol.* 291 67–78. 10.1152/ajprenal.00412.2005 16478975

[B19] Li J. Bonkowski M. S. Moniot S. Zhang D. Hubbard B. P. Ling A. J. (2017). A conserved NAD(+) binding pocket that regulates protein-protein interactions during aging. *Science* 355 1312–1317. 10.1126/science.aad8242 28336669 PMC5456119

[B20] Lynch M. R. Tran M. T. Ralto K. M. Zsengeller Z. K. Raman V. Bhasin S. S. (2019). TFEB-driven lysosomal biogenesis is pivotal for PGC1alpha-dependent renal stress resistance. *JCI Insight* 5:e126749. 10.1172/jci.insight.126749 30870143 PMC6538327

[B21] Mills K. F. Yoshida S. Stein L. R. Grozio A. Kubota S. Sasaki Y. (2016). Long-term administration of nicotinamide mononucleotide mitigates age-associated physiological decline in mice. *Cell. Metab.* 24 795–806. 10.1016/j.cmet.2016.09.013 28068222 PMC5668137

[B22] Pham T. X. Bae M. Kim M. B. Lee Y. Hu S. Kang H. (2019). Nicotinamide riboside, an NAD+ precursor, attenuates the development of liver fibrosis in a diet-induced mouse model of liver fibrosis. *Biochim. Biophys. Acta Mol. Basis Dis.* 1865 2451–2463. 10.1016/j.bbadis.2019.06.009 31195117 PMC6614025

[B23] Ratliff B. B. Abdulmahdi W. Pawar R. Wolin M. S. (2016). Oxidant mechanisms in renal injury and disease. *Antioxid Redox Signal* 25 119–146. 10.1089/ars.2016.6665 26906267 PMC4948213

[B24] Rewa O. Bagshaw S. M. (2014). Acute kidney injury-epidemiology, outcomes and economics. *Nat. Rev. Nephrol.* 10 193–207. 10.1038/nrneph.2013.282 24445744

[B25] Rogakou E. P. Pilch D. R. Orr A. H. Ivanova V. S. Bonner W. M. (1998). DNA double-stranded breaks induce histone H2AX phosphorylation on serine 139. *J. Biol. Chem.* 273 5858–5868. 10.1074/jbc.273.10.5858 9488723

[B26] Sharma A. Singh K. Almasan A. (2012). Histone H2AX phosphorylation: a marker for DNA damage. *Methods Mol. Biol.* 920 613–626. 10.1007/978-1-61779-998-3_40 22941631

[B27] Small D. M. Morais C. Coombes J. S. Bennett N. C. Johnson D. W. Gobe G. C. (2014). Oxidative stress-induced alterations in PPAR-gamma and associated mitochondrial destabilization contribute to kidney cell apoptosis. *Am. J. Physiol. Renal Physiol.* 307 814–822. 10.1152/ajprenal.00205.2014 25122050

[B28] Tran M. T. Zsengeller Z. K. Berg A. H. Khankin E. V. Bhasin M. K. Kim W. (2016). PGC1alpha drives NAD biosynthesis linking oxidative metabolism to renal protection. *Nature* 531 528–532. 10.1038/nature17184 26982719 PMC4909121

[B29] Tsubota K. (2016). The first human clinical study for NMN has started in Japan. *NPJ Aging Mech. Dis.* 2:16021. 10.1038/npjamd.2016.21 28721273 PMC5515004

[B30] Tsuruya K. Furuichi M. Tominaga Y. Shinozaki M. Tokumoto M. Yoshimitsu T. (2003). Accumulation of 8-oxoguanine in the cellular DNA and the alteration of the OGG1 expression during ischemia-reperfusion injury in the rat kidney. *DNA Repair (Amst)* 2 211–229. 10.1016/s1568-7864(02)00214-8 12531391

[B31] Us National Library of Medicine. (2014). *ClinicalTrials.gov.* Available Online at: https://clinicaltrials.gov/ct2/show/NCT02191462.

[B32] Us National Library of Medicine. (2015). *ClinicalTrials.gov.* Available Online at: https://clinicaltrials.gov/ct2/show/NCT02300740.

[B33] Us National Library of Medicine. (2016a). *ClinicalTrials.gov.* Available Online at: https://clinicaltrials.gov/ct2/show/NCT02689882.

[B34] Us National Library of Medicine. (2016b). *ClinicalTrials.gov.* Available Online at: https://clinicaltrials.gov/ct2/show/NCT02303483.

[B35] Us National Library of Medicine. (2016c). *ClinicalTrials.gov.* Available Online at: https://clinicaltrials.gov/ct2/show/NCT02835664.

[B36] Us National Library of Medicine. (2017). *ClinicalTrials.gov.* Available Online at: https://clinicaltrials.gov/ct2/show/NCT02712593.

[B37] Valentijn F. A. Falke L. L. Nguyen T. Q. Goldschmeding R. (2018). Cellular senescence in the aging and diseased kidney. *J. Cell. Commun. Signal* 12 69–82. 10.1007/s12079-017-0434-2 29260442 PMC5842195

[B38] Varrier M. Forni L. G. Ostermann M. (2015). Long-term sequelae from acute kidney injury: potential mechanisms for the observed poor renal outcomes. *Crit. Care* 19:102. 10.1186/s13054-015-0805-0 25887052 PMC4361133

[B39] Yang L. (2019). How acute kidney injury contributes to renal fibrosis. *Adv. Exp. Med. Biol.* 1165 117–142. 10.1007/978-981-13-8871-2_7 31399964

[B40] Yang L. Besschetnova T. Y. Brooks C. R. Shah J. V. Bonventre J. V. (2010). Epithelial cell cycle arrest in G2/M mediates kidney fibrosis after injury. *Nat. Med.* 16 535–543. 10.1038/nm.2144 20436483 PMC3928013

[B41] Yang L. Xing G. Wang L. Wu Y. Li S. Xu G. (2015). Acute kidney injury in china: a cross-sectional survey. *Lancet* 386 1465–1471. 10.1016/S0140-6736(15)00344-X 26466051

[B42] Zheng M. Cai J. Liu Z. Shu S. Wang Y. Tang C. (2019). Nicotinamide reduces renal interstitial fibrosis by suppressing tubular injury and inflammation. *J. Cell. Mol. Med.* 23 3995–4004. 10.1111/jcmm.14285 30993884 PMC6533567

[B43] Zhu R. Wang W. Yang S. (2019). Cryptotanshinone inhibits hypoxia/reoxygenation-induced oxidative stress and apoptosis in renal tubular epithelial cells. *J. Cell. Biochem.* 120 13354–13360. 10.1002/jcb.28609 30891815

[B44] Zhu S. Pabla N. Tang C. He L. Dong Z. (2015). DNA damage response in cisplatin-induced nephrotoxicity. *Arch. Toxicol.* 89 2197–2205. 10.1007/s00204-015-1633-3 26564230 PMC4734632

