# Supplementary figures and images for: Nicotinamide Mononucleotide Attenuates Renal Interstitial Fibrosis After AKI by Suppressing Tubular DNA Damage and Senescence
Source: Front Physiol. 2021 Mar 23;12:649547. doi: 10.3389/fphys.2021.649547 (PMC8021789; doi:10.3389/fphys.2021.649547)

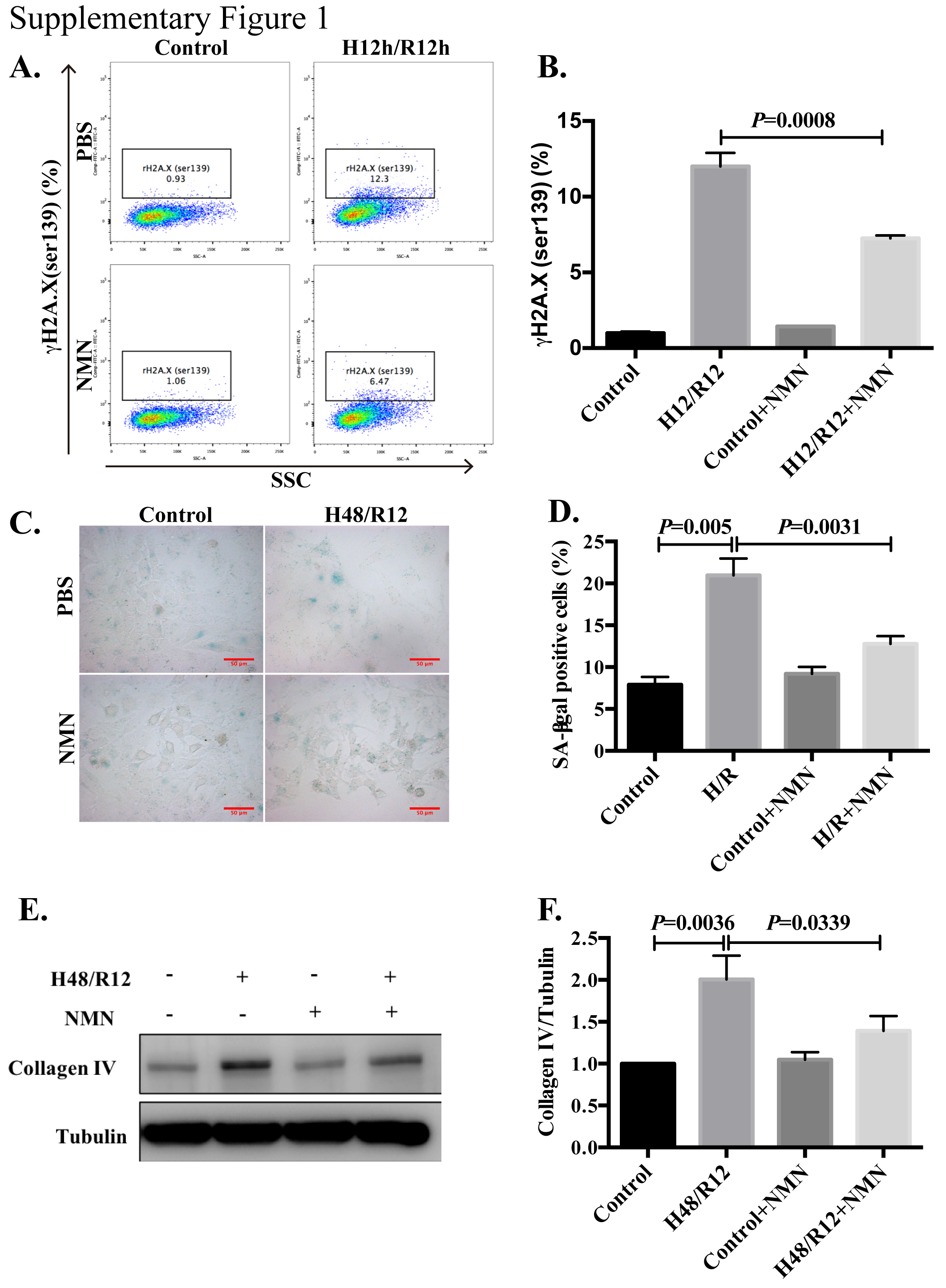

Supplement: Supplementary file 1 [file Image_1.tif]

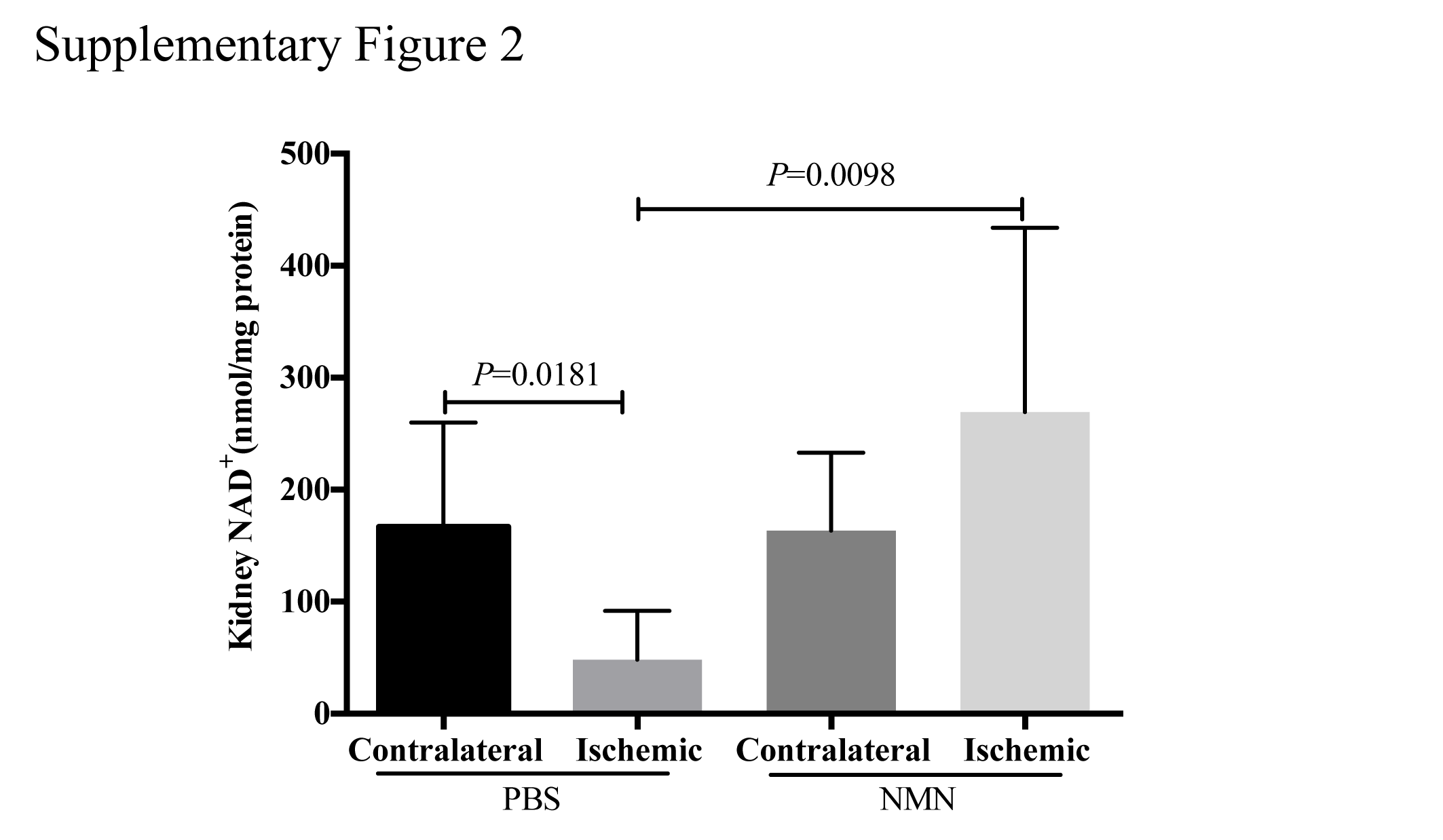

Supplement: Supplementary file 2 [file Image_2.tif]
