# Supplementary material for: Nicotinamide Mononucleotide Attenuates Renal Interstitial Fibrosis After AKI by Suppressing Tubular DNA Damage and Senescence
Source: Front Physiol. 2021 Mar 23;12:649547. doi: 10.3389/fphys.2021.649547 (PMC8021789; doi:10.3389/fphys.2021.649547)
Supplement: Supplementary file 3 [file Presentation_1.pdf]

Captions for supplementary figures:

Supplementary Figure 1. Nicotinamide Mononucleotide (NMN) attenuated hypoxia-reoxgenation induced injuries in HK-2 cells. (A-B) Flow cytometrey analysis of γH2A.X(ser139) of HK-2 cells after subjected to hypoxia for 12 hours followed by 12 hours reoxgenation (H12/R12), and bar graph showed decreased DNA damage in NMN-treated group. (C-D) Decreased percentage of SA-β-gal positive cells in NMN-treated group after subjected to hypoxia for 48 hours followed by 12 hours reoxgenation (H48/R12). (E-F) Western blots analysis showed reduction of collagen IV in NMN-treated groups after 48 hours hypoxia followed by 12 hours reoxgenation (H48/R12).

Supplementary Figure 2. NAD^+ levels in the contralateral and ischemic kidney tissues of uIRI mice given PBS or NMN. n=6/group. data are means±SD.
